# The promising roles of macrophages in geriatric hip fracture

**DOI:** 10.3389/fcell.2022.962990

**Published:** 2022-08-26

**Authors:** Yi-ning Lu, Ling Wang, Ying-ze Zhang

**Affiliations:** ^1^ Department of Orthopedic Research Center, The Third Hospital of Hebei Medical University, Shijiazhuang, China; ^2^ Department of Orthopedic Surgery, The Third Hospital of Hebei Medical University, Shijiazhuang, China; ^3^ Department of Orthopedic Oncology, The Third Hospital of Hebei Medical University, Shijiazhuang, China

**Keywords:** aging, macrophages, hip fracture, healing, immune

## Abstract

As aging becomes a global burden, the incidence of hip fracture (HF), which is the most common fracture in the elderly population and can be fatal, is rapidly increasing, and its extremely high fatality rate places significant medical and financial burdens on patients. Fractures trigger a complex set of immune responses, and recent studies have shown that with aging, the immune system shows decreased activity or malfunctions in a process known as immune senescence, leading to disease and death. These phenomena are the reasons why elderly individuals typically exhibit chronically low levels of inflammation and increased rates of infection and chronic disease. Macrophages, which are key players in the inflammatory response, are critical in initiating the inflammatory response, clearing pathogens, controlling the innate and adaptive immune responses and repairing damaged tissues. Tissue-resident macrophages (TRMs) are widely present in tissues and perform immune sentinel and homeostatic functions. TRMs are combinations of macrophages with different functions and phenotypes that can be directly influenced by neighboring cells and the microenvironment. They form a critical component of the first line of defense in all tissues of the body. Immune system disorders caused by aging could affect the biology of macrophages and thus the cascaded immune response after fracture in various ways. In this review, we outline recent studies and discuss the potential link between monocytes and macrophages and their potential roles in HF in elderly individuals.

## Introduction

Hip fracture (HF), mainly including intertrochanteric and femoral neck fracture, is considered to be the most common fracture in elderly patients and imposes a significant global health burden as the population ages (Collin et al., 2017). Accumulating evidence indicates that the incidence of HF is estimated to increase from 1.9 million in 1990 to 6.26 million in 2050 (Cooper et al., 2011). HF has a very high fatality rate; the 1- and 2-year mortality rates for HF among elderly individuals have been reported to be as high as 29%, and 38%, respectively, which are much higher than those of other types of fractures among geriatric patients (Yin et al., 2015). Reduced quality of life, subsequent secondary fracture and heavy financial burdens make HF an increasingly important public health concern for elderly individuals. Healthy older individuals experience higher levels of stress than young adults, and HF in elderly patients is associated with complicated immunological changes, including changes in innate proinflammatory phenotypes and adaptive immunosuppressive responses (Vallet et al., 2020). In addition, studies have shown that geriatric HF patients have a reduced ability to exhibit an effective antimicrobial immune response and that these immunological effects can be sustained until 7 days after surgery (Sutherland et al., 2015). As important immune cells, macrophages play key roles in not only the immune system but also bone formation and healing after trauma (Gu et al., 2017).

Macrophages are central players in trauma-induced innate and adaptive immune responses (Oishi and Manabe, 2018). In addition to their roles in sensing tissue damage and initiating inflammation, macrophages critically contribute to tissue repair and wound healing (Peiseler and Kubes, 2018). There is a growing body of evidence showing that macrophages undergo important functional alterations that affect multiple stages after fracture (McCauley et al., 2020; Zhao et al., 2020; Horibe et al., 2021). Macrophages not only participate in the first phase of inflammation but also secrete proangiogenic and mitogenic factors to promote the progression of the other three phases, including fibrocartilage formation (early anabolic phase), hard callus formation (late anabolic phase) and the late phase of tissue remodeling (Ono and Takayanagi, 2017; Schlundt et al., 2018). Understanding the roles and mechanisms of macrophages in the context of fracture may be helpful in predicting the timing of surgery for geriatric HF patients and reducing the immunosuppressive effects that lead to mortality. Macrophages are widely active in all types of fracture populations (including populations of different ages and with fractures at different sites) and play a significant role in all phases of the postinjury period. HF is the most common in the elderly population and has the poorest prognosis. As the *in vivo* microenvironment ages, macrophages undergo dramatic changes in function and phenotype. Thus, our discussion will focus on the changes in macrophage function and phenotype in elderly HF patients and the significance of these changes. This review will outline recent studies and discuss the potential link between monocytes and macrophages and their potential roles in HF in elderly individuals.

## Characteristics of macrophages in elderly individuals

In 1882, Metchnikov et al. first identified macrophages as an immune subgroup and gradually discovered the immune functions of macrophages, as reported in 1908. He proposed that phagocytes, such as macrophages, monocytes and neutrophils, could fight pathogens, for which he was awarded the Nobel Prize in Physiology or Medicine (Cavaillon, 2011).

### Origin and polarization

It is now generally accepted that there are two different sources of tissue macrophages. Most tissue-resident macrophages (TRMs) can be traced to the yolk sac stage at E8.5/E9.0 and do not necessarily require a blood supply (McGrath et al., 2003). During inflammatory resolution, TRMs can clear apoptotic cells, proteins, and phospholipids and remove some particles and pathogens in response to toxins in the local microenvironment (Davies et al., 2013). Many TRMs are able to maintain themselves through local proliferation without the contribution of monocyte-derived macrophages (Schulz et al., 2012). TRMs produce a variety of factors that stimulate the activation, proliferation and differentiation of immune cells, epithelial cells, endothelial cells (ECs), fibroblasts and stem cells, promoting tissue homeostasis (Watanabe et al., 2019). As another source, macrophages can develop from monocytes that infiltrate tissues during adulthood, and these cells mediate the major pathological inflammatory response, especially upon encountering an acute exogenous injury (Sreejit et al., 2020). Bone marrow monocytes are recruited into the injured tissue, where they differentiate into macrophages. During injury, TRMs and monocyte-derived macrophages play different roles. Monocyte-derived macrophages generally exhibit stronger anti-inflammatory functions than TRMs (Sreejit et al., 2020). Both types of macrophages seem to coexist in tissues and sustain a stable physiological environment (Sreejit et al., 2020).

Macrophage polarization occurs in a specific time and space. It is a dynamic process stimulated by changes in the microenvironment to produce different functional phenotypes (Chen et al., 2021). Macrophages are broadly classified into three subsets, namely, primary macrophages (M0), classically activated (M1) macrophages, and alternatively activated (M2) macrophages. M0 macrophages can polarize to M1-or M2-type macrophages under different conditions (Miao et al., 2017). M1 macrophages are usually activated by lipopolysaccharide (LPS), interferon gamma (IFN-γ) and a combination of other cytokines, such as granulocyte-macrophage colony-stimulating factor (GM-CSF) and tumor necrosis factor *a* (TNF-α). Interleukin 4 (IL-4), interleukin 10 (IL-10), interleukin 13D (IL-13D) and transforming growth factor *ß* (TGF-β) induce the activation of M0 macrophages to M2 macrophages (Sica et al., 2015). The M1/M2 macrophage nomenclature was inspired by the T-helper 1 (Th1) vs. T-helper 2 (Th2) concept introduced by Mosmann and Coffman (Mosmann et al., 1986). Although it has been widely used, many experts in this field still have many conflicting views on macrophage typing (Hume, 2015; Nahrendorf and Swirski, 2016; Murray, 2017). Indeed, M1 and M2 macrophages represent opposing final forms of macrophages, and other groups of macrophages exist in between. Shivshankar et al. demonstrated that caveolin-1 also seems to play an important role in the activation of M2 macrophages (Shivshankar et al., 2014). According to the different stimuli, M2 macrophages can be divided into four subtypes: the M2a-like, M2b-like, M2c-like and M2d-like subtypes (Atri et al., 2018). M2a-like macrophages are stimulated by IL-4 or IL-13 and express high levels of CD86 and CD200R and low levels of CD14 and Toll-like receptor 4 (TLR4); M2b-like macrophages are stimulated by LPS or IL-1β and are characterized by high CD80 and CD14 expression together with high production of IL-10, C-C motif chemokine ligand 1 (CCL1) and proinflammatory cytokines; M2c-like macrophages are stimulated by IL-10 and produce C-C motif chemokine ligand 18 (CCL18) and C-C motif chemokine ligand 16 (CCL16); M2d-like macrophages can suppress proinflammatory M1 macrophages and are mainly characterized by high IL-10 production, low IL-12 and TGF-β production and C-X-C motif chemokine ligand 10 (CXCL10), C-X-C motif chemokine ligand 16 (CXCL16) and C-C motif chemokine ligand 5 (CCL5) chemokine secretion. Several studies have focused on human macrophage surface markers, providing an important reference for determining the *in vitro* activation status of human macrophages and their potential roles in the immune response (Tarique et al., 2015; Chernykh et al., 2018). Regulatory macrophages (MRs) are a subtype of macrophages with immunosuppressive and anti-inflammatory properties. They are mainly derived from bone marrow precursor cells and play a key role in limiting innate and adaptive immune responses (Zhang et al., 2021). It has been shown that LPS can stimulate MRs to produce large amounts of IL-10 and constitute a novel type of suppressor macrophage involved in the suppression of alloimmune responses *in vitro* (Suzuki et al., 2016; Guo et al., 2017). MRs highly express major histocompatibility complex II (MHC-II) and CD80 and can secrete IL-10 and TGF-β as well as a small amount of IL-12 (Mosser and Edwards, 2008). Erbel et al. named a new macrophage phenotype, M4 macrophages, induced by the platelet chemokine C-X-C motif chemokine ligand 4 (CXCL4), and their study suggested that M4 macrophages contribute to atherosclerotic plaque and oxidative lesion development by promoting the accumulation of oxidative low-density lipoprotein (LDL) in phagocytic cells (Erbel et al., 2015). De Sousa et al. found that M4 macrophages are also one of the cell types involved in the microbial response to leprosy (de Sousa et al., 2018). The process of macrophage polarization is complex, and many unknown factors are involved in the regulation of macrophage polarization. For example, protease-activated receptor 2 (PAR2) activation induces M1 polarization and inflammation, which may explain the promotion of macrophage-associated inflammation in adipose tissue and atherosclerotic plaques by mast cell trypsin (Chen et al., 2019). Signal transducer and activator of transcription (STAT) proteins also appear to be key factors in M1 and M2 macrophage polarization. The mutual exclusivity of these signaling pathways may be a key factor in M1 and M2 polarization (Lawrence and Natoli, 2011). The recognition of polarization states and targeted regulation of macrophage polarization offer the possibility of treating acute and chronic inflammatory diseases.

Generally, M1 macrophages can produce large amounts of proinflammatory cytokines (including IFN-γ, IL-1β, IL-6, IL-12 and TNF-α) during immune activation to induce Th1 responses. With the need to regulate local tissue repair, M1 macrophages can effectively polarize into M2 macrophages (Klopfleisch, 2016), expressing different genes and attaining distinct functional states. M2 macrophages are anti-inflammatory and antiparasitic and can exert a suppressive effect on Th1 cell-mediated type 1 inflammation. M2 macrophages can produce cytokines, including IL-10 and TGF-β, thus inducing Th2 immune responses and contributing to angiogenesis, wound healing progression and granulation tissue maturation. Moreover, the archetypal Th2 cytokine IL-4 is a fundamental component that drives the accumulation of tissue macrophages through self-renewal (Jenkins et al., 2011). Fracture repair could be completed under the condition that proinflammatory cytokines in the microenvironment transition to anti-inflammatory cytokines to maximize the regenerative and minimize the destructive effects even in the absence of T and B cells (Toben et al., 2011). However, the precise mechanisms that regulate M1 and M2 macrophage polarization and functions in geriatric trauma have still not been fully elucidated. In addition, there are also a special subgroup of bone-resident macrophages, termed “osteomacs”. Osteomacs account for approximately one-sixth of all cells in bone marrow and reside within bone as a distinctive canopy structure overlying mature osteoblasts (Chang et al., 2008). Osteomacs have been proven to play an important role in fracture repair and bone remodeling (Parfitt, 2001; Chang et al., 2008; Raggatt et al., 2014; Michalski et al., 2019).

### Fundamental functions

Macrophages serve many different functions in the immune system and play important roles in defending against pathogenic attacks. As the main factors in the inflammatory response, macrophages participate in the immune response by releasing a large number of inflammatory mediators (Gordon, 2007). Macrophages are capable of presenting antigens, activating T and B cells, and directly stimulating adaptive immune responses. In addition, macrophages can maintain tissue homeostasis by removing apoptotic cells and producing growth factors. Furthermore, macrophages are essential for maintaining a balanced response to tissue damage signals. When this balance is disturbed, the inflammatory cascade ensues. In trauma-induced aseptic inflammation, danger-associated molecular patterns (DAMPs) are activated, and endogenous necrotic or stressed cells trigger an inflammatory response to initiate macrophage activity. After macrophages are activated, proinflammatory cytokines such as TNF-α, IL-1β and INF-γ are released (Zhang and Wang, 2014), and inflammatory monocytes are recruited from the bone marrow and converted into monocyte-derived macrophages. Tissue macrophages and newly generated macrophages undergo phenotypical and functional changes (e.g., an enhanced TLR4 response and antimicrobial response) (Stout and Suttles, 2004) to help alleviate inflammation and mediate tissue repair by secreting growth factors and anti-inflammatory cytokines (Hirsiger et al., 2012). Tissue injury also results in an influx of monocytes into the blood, and some of the recruited monocytes travel to the affected injury site and differentiate into macrophages (Hirsiger et al., 2012). Macrophages play an indispensable role in tissue repair. Many growth factors secreted by macrophages (e.g., fibroblast growth factor (FGF), platelet growth factor (PDGF), and prostaglandins) promote vascular growth, fibroblast proliferation and collagen deposition, which in turn promote tissue remodeling and regeneration (Berse et al., 1992; Chujo et al., 2009; Willenborg et al., 2012; Wynn and Vannella, 2016). Several researchers have identified other mediators and cells involved in the macrophage-mediated repair process, including cytokines (e.g., IL-4 and IL-13), regulatory T cells (Tregs), and B1B cells (Mosser and Edwards, 2008; Wynn and Ramalingam, 2012). Tissue inhibitor of metalloproteinases 1 (TIMP1) is also produced by macrophages to counteract the effects of matrix metalloproteinases (MMPs) and promote the formation of extracellular matrix (ECM) (Murray and Wynn, 2011). The resolution of inflammation and the metabolic phase of fracture healing require sufficient progenitor cells, suggesting that progenitor cells are essential for fracture healing (Gerstenfeld et al., 2003), and macrophages have been shown to influence the local microenvironment of progenitor cells. In addition, macrophages producing IL-10 have been shown to have a very strong effect on progenitor cell renewal (London et al., 2011).

### Changes in macrophage numbers and phenotypes with age

Aging is a complicated pathophysiological process accompanied by a wide array of biological adaptations and is often associated with the onset of many pathologies. Immunosenescence, which refers to the gradual deterioration of the immune system with age, represents an important feature of the aging process. Immunosenescence reflects the effects of age-related changes on the immune system, both cellular and serological, as well as processes that produce specific responses to foreign and self-antigens. Many findings suggest that macrophages, which are members of the innate immune system, undergo various changes with age, which may explain the posttraumatic manifestations in specific populations, such as elderly individuals (Li M. et al., 2021; Esfahani et al., 2021). We will discuss recent progress in understanding the roles of macrophages in the context of aging.

Connie et al. discovered that the number of macrophages in the spleen and bone marrow of aged mice was much higher than that in young mice; moreover, the phenotypes of macrophages were quite different between the two groups (Jackaman et al., 2013). Macrophages in elderly individuals may be skewed toward a more anti-inflammatory and proangiogenic phenotype. Interestingly, several studies have seemed to draw the opposite conclusions, as Clark et al. showed delayed fracture healing and reduced bone mass in aged mice and upregulation of M1/proinflammatory genes in macrophages of aged mice (Clark et al., 2020). Furthermore, Mahbub et al. showed an age-related defect in M1 and M2 responses to abnormal further polarization in aged mice compared to young mice, as indicated by reduced expression or production of the M1 markers inducible nitric oxide synthase (iNOS), IL-6, IL-1β and TNF-α and the M2 markers arginase 1 (Arg1), found in inflammatory zone 1 (FIZZ1) and Ym1, suggesting that the reduction in the ability to undergo classic M1 or M2 polarization could be attributed to the aging microenvironment rather than to the macrophages themselves (Mahbub et al., 2012). The number of macrophages located at different tissue sites changes with aging. The M2 phenotype tends to be predominant in healthy human skeletal muscle, and both the absolute numbers and percentage of total macrophages increase with age. However, M1 macrophages show lower abundance in human skeletal muscle that decreases with age (Cui et al., 2019) and in some age-related liver diseases (e.g., liver fibrosis) (Mahrouf-Yorgov et al., 2011). In addition, Franceschi et al. demonstrated that macrophages could augment inflammatory responses upon activation by recognizing DAMPs or alarmins (Franceschi et al., 2017). However, the mechanisms of the progressive age-induced redistribution and activation of macrophages and macrophage-derived cells throughout the body remain unclear.

### Alterations in cytokine and chemokine secretion by macrophages with aging

Coordination of the inflammatory response is the main function of macrophages. In the immune response, macrophages secrete a wide range of cytokines, chemokines, growth factors and enzymes in response to pathogens and danger signals (Liu et al., 2014). As aging progresses, alterations in the immune system, dysregulation of chemokine and cytokine secretion and changes in the expression of functional chemokine and cytokine receptors may occur and result in poor responses to infection (Shaw et al., 2010).

TLRs are a subset of pattern recognition receptors (PRRs) and a major class of receptors expressed by macrophages. Macrophages can express all TLRs but mainly express TLRs 1, 2, 4, 5, and 8 (Mahbub et al., 2012). TLRs recognize viral nucleic acids and bacterial products, including LPS and lipo-lactic acid (LTA), as well as endogenous ligands, and can be activated by these factors (Kawai and Akira, 2010). In the early stages of inflammation, the activation of TLRs on macrophages leads to the production of inflammatory cytokines such as TNF-α, IL-1β and IL-6, all of which play irreplaceable roles in the inflammatory response (Linehan and Fitzgerald, 2015). However, at a later stage, TLRs have been shown to signal the removal of cellular debris and the restoration of tissue homeostasis (Wissinger et al., 2009). TLR2 is not only expressed by a very high proportion of macrophages but is also important for activating macrophage function, especially pathogen-induced activation and maturation during inflammation (Huang et al., 2007; Arleevskaya et al., 2020). TLR4 on the cell surface propagates signals through MyD88-dependent and -independent pathways, leading to the production of proinflammatory cytokines and type I interferons, respectively (Ballinger et al., 2010; Herold et al., 2011). In addition, TLR4 induces the formation of inflammatory vesicles that are capable of controlling inflammatory responses and coordinating antimicrobial host defense through the induction of the cell membrane innate immune sensor NOD-like receptor family pyrin domain-containing 3 (NLRP3), adaptor apoptosis-associated speck-like protein containing a caspase recruitment domain (ASC), and caspase 1 (de Zoete et al., 2014; Vanaja et al., 2016). Recently, it has been demonstrated that TLR4 may be a target receptor for exosomes that induce an inflammatory response in macrophages (Kojima et al., 2018). Experiments have shown that aging alters TLR-induced cytokine secretion by macrophages and that TLR expression by splenic and thioglycolate-induced peritoneal macrophages changes (Plowden et al., 2004). Boehmer et al. reported reductions in LPS-induced IL-6 and TNF-α secretion in aged murine thioglycolic acid-induced peritoneal macrophages and decreased LPS-induced secretion of IL-6, TNF-α, IL-1β and IL-12 in splenic macrophages in aged mice, accompanied by increased secretion of IL-10 (Boehmer et al., 2004). However, in contrast to splenic macrophages, aged microglia secreted more proinflammatory cytokines in response to TLR stimulation, indicating differences between different tissue macrophages with aging (Hoogland et al., 2015). The decline in TLR functions inevitably affects the inflammatory functions of macrophages by changing their pattern recognition ability. The defects and dysfunction in TLR expression may increase susceptibility to viruses and bacteria and the severity of posttraumatic complications in elderly individuals. These findings may explain why older patients often fail to exhibit an adequate immune response to infection or immunity. In addition to cytokines, chemokines, and IFNs, TLRs can upregulate the expression of hundreds of genes in macrophages (Takeuchi and Akira, 2010).

IFN-γ is an important cytokine involved in host protection against intracellular pathogens and immunopathology after infection, coordinates many different cellular programs and signaling, activates macrophages and leads to the killing of intracellular pathogens (Thakur et al., 2019). Helen Vest et al. demonstrated that IFN-γ concentrations were significantly lower in older patients than in younger patients after trauma or surgery (Vester et al., 2014). In addition to immune damage, it has been shown that decreased IFN-γ expression in macrophage subpopulations lead to satellite cell dysfunction in aged skeletal muscle (Zhang C. et al., 2020). Herrero et al. has shown that macrophages from older mice express 50% fewer MHC-II molecules on the cell surface after IFN-γ stimulation, which could lead to decreased macrophage antigen presentation activity compared with that of macrophages from young mice (Herrero et al., 2001).

Other studies have shown that the expression of cyclooxygenase 2 (COX2) and subsequent production of prostaglandin 2 (PGE2) are much higher in aged macrophages than in younger cells (Hayek et al., 1997; Chen et al., 2013), suggesting that COX2 expression may be associated with increased expression of inflammatory cytokines such as TNF-α and IL-6. The increased level of COX2 is thought to be due to an increase in the transcription rate rather than in transcript stability (Claycombe et al., 2002). These findings may facilitate the development of therapeutic interventions to inhibit or delay age-associated dysregulation of the immune and inflammatory responses. Aging can significantly affect macrophages; however, these cells are affected by the microenvironment, and the mechanism underlying the impairment of macrophage functions is still unclear.

### Changes in macrophage functions

Aging is one of the most intricate and complex biological processes and can influence not only the phenotype and cytokine secretion but also the major biological functions of macrophages. The phagocytic capacity of macrophages declines and reduces the clearance of apoptotic cells in aged mice due to decreased phagocytosis, resulting in the accumulation of apoptotic debris, which can contribute to immune system dysfunction and chronic inflammatory responses (Aprahamian et al., 2008). This defect, which causes persistent inflammation, is present in humans (De Maeyer et al., 2020). The decline in phagocytosis is thought to be an important factor in the pathogenesis of systemic lupus erythematosus. Moreover, age-associated impairments in phagocytosis can vary depending on tissue distribution. Some findings have suggested that alveolar macrophage phagocytosis does not decline with age; however, some essential functions of peritoneal macrophages decrease (Mancuso et al., 2001). The decline in peritoneal macrophage phagocytosis might be attributed to age-related changes in the peritoneal microenvironment rather than intrinsic defects in macrophages; therefore, it may be reversible and become a therapeutic target (Linehan and Fitzgerald, 2015).

Senescent cells play complex roles during normal wound healing and in chronic wounds. These cells can interfere with the process of wound repair and tissue regeneration in humans and rodents. Swift et al. strongly suggested that delayed wound healing was significantly associated with age-associated changes in the functions of macrophages (Swift et al., 1999). Macrophages in aged mice produce less vascular endothelial growth factor (VEGF) than those in young mice (Swift et al., 1999). Fracture repair is induced by a large number of immune cells and an inflammatory cascade response. The adaptive immune system changes with age due to repeated exposure to pathogens/antigens (Kverneland et al., 2016). The altered early healing response caused by these changes in the adaptive immune system due to aging leads to impaired bone repair, delayed healing, and even the development of osteonecrosis. Macrophages can promote angiogenesis and granulation tissue maturation, in addition to regulating pro- and anti-inflammatory signaling balance. Thus, aging-induced alterations in macrophage functions may affect bone repair at different stages and age-related defects in bone regeneration. Data from Loffler et al. showed an overall reduction in the expression of the monocyte/panmacrophage markers CD14 and CD68 in aged rats and attenuated expression of anti-inflammatory M2 macrophage markers in the hematomas of aged animals, which was associated with reduced vascular regeneration of the bone callus. With the local transplantation of CD14 ^+^ macrophage precursors into aged rats with fractures, a significant induction of new bone tissue deposition, a reduction in fibrosis and a marked improvement in callus vascularization were observed (Löffler et al., 2019). These results suggested that the related impairment in bone regeneration was due to impaired macrophage function (Löffler et al., 2019). Moreover, Clark et al. have demonstrated that macrophages from old mice have a stronger M1 proinflammatory gene signature than macrophages from young mice, which adversely affects the outcome of fracture healing (Clark et al., 2020). Based on this result, they attempted to inhibit the recruitment of macrophages from bone marrow using the M-CSF-1R inhibitor PLX3397 during the bone healing phase. Inhibiting macrophage recruitment in aged mice proved to be beneficial for fracture healing (Clark et al., 2020). Additionally, Naik et al. observed that COX-2 expression was reduced in aging mice during the early inflammatory phase of bone repair, which could be another factor contributing to delayed bone remodeling (Naik et al., 2009).

With the advancement of bone modeling, the number of mesenchymal stem cells (MSCs) in the bone marrow decreases, along with their ability to undergo osteogenic differentiation (Gibon et al., 2016b). Yin et al. cocultured MSCs from young and old mice with RAW 264.7 macrophages and found that young MSCs increased the mRNA expression of M2-associated Arg1 and IL-10, while aged MSCs increased the mRNA expression of M1-associated TNF-α (Yin et al., 2017). Lee et al. transplanted MSCs into the wounds of young and aged mice and showed that activated MSCs restored the regenerative process and reversed the effects of aging in aged mice (Lee et al., 2013). Although a large number of studies have shown that aging affects the physiology of macrophages and MSCs, the mechanisms by which aging affects these cells are still poorly understood (Choudhery et al., 2012; Bernet et al., 2014). Aging seems to be increasingly related to macrophage-mediated fracture healing delay, but some studies have presented new perspectives on this subject. A study by Blacher et al. demonstrated that the circadian rhythm of macrophage function and macrophage transcriptional responses is significantly disrupted with age (Blacher et al., 2022). A study reported by Clark et al. demonstrated significant differences in the transcriptome of macrophages from fracture calluses in aged and young mice, with upregulated expression of M1/proinflammatory genes and dysregulated expression of other immune-related genes in aged mouse macrophages (Clark et al., 2020). These phenomena are related to the accumulation of genetic changes over time, and the loss of macrophage autophagy leads to multiple functional changes that are associated with macrophage aging (Stranks et al., 2015). However, the exact mechanism by which aging leads to a decrease in autophagy remains unclear.

## Role of macrophages in geriatric HF

### HF in elderly individuals

HFs in the elderly are very serious injuries. According to statistics, only 37% of HF patients recover to their prefracture physiological status, which can lead to high medical costs and heavy social burdens on families (Lui et al., 2015). Aging considerably increases the risk of falls, with alarmingly high rates of falls in adults over 65 years of age, resulting in up to 30% of serious HFs (Inouye et al., 2009). Therefore, HFs are considered to account for the majority of all types of fractures in elderly patients (Rogmark et al., 2018). In the inflammatory phase after fracture, activated macrophages produce proinflammatory mediators, as well as multiple tissue-degrading enzymes that exacerbate the inflammatory environment and lead to cartilage and bone destruction (Zhang H. et al., 2020). Studies have shown that macrophage polarization is closely related to osteogenesis. The imbalance in macrophage polarization caused by aging is one of the essential causes of osteoporosis (Munoz et al., 2020). Macrophages affect osteoclasts, osteoblasts, and osteocytes during bone loss; thus, adequate macrophage inhibition improves systemic inflammation associated with osteoporosis (Yang and Yang, 2019). As a common geriatric disease, osteoporosis is one of the main causes of HF in elderly individuals; furthermore, the macrophage dysfunction that occurs with aging delays the fracture healing process. Therefore, understanding this phenomenon and discovering the underlying mechanism are critical for treating geriatric HF patients.

### Macrophages in the posttraumatic phase

The function of macrophages in the posttraumatic inflammatory response is multifaceted. As the first line of defense against invading microorganisms, macrophages are able to respond rapidly to exogenous dangers and become activated. Trauma and tissue damage (mainly cell necrosis and loss of plasma membrane integrity) lead to the release of endogenous cellular DAMPs, which are known as alarmins. These factors are mainly released from damaged cells (Lotze et al., 2007) and activate immune cells through TLR signaling (Peiseler and Kubes, 2018). DAMPs lead to immune responses by activating PRRs on immune cells, which were originally described in the context of immune responses to microorganisms and are activated by pathogen-associated molecular patterns (PAMPs) in response to infection (Thompson et al., 2011). Heat shock protein (Hsp), high mobility group box protein 1 (HMGB1), IL-1α and IL-33 are representative DAMPs that trigger PRRs and activate macrophages (Chen and Nuñez, 2010); additionally, macrophages are capable of detecting tissue damage. Activated macrophages release proinflammatory cytokines (such as TNF-α, IL-1β and IFN-γ), recruit immune cells to the site of injury, and then participate in the immune response by phagocytosing cellular debris and secreting proinflammatory cytokines (Zhang and Mosser, 2008). The recruitment of monocytes to sites of inflammation is critical for host defense, and this process depends primarily on CCR2. During inflammation, monocytes circulate in the bloodstream and extravasate to inflamed tissues after a general leukocyte recruitment cascade (including rolling, adhesion and migration) (Kratofil et al., 2017). Macrophages with a proinflammatory phenotype are activated by infection and produce a variety of proinflammatory mediators, including TNF-α, IL-1, IL-6 and IFN-γ, which are involved in the activation of multiple microbicidal mechanisms and contribute to the clearance of invading pathogens. Activated macrophages can promote the polarization of Th1 and Th17 cells by producing IL-12 and IL-23 or support the differentiation of Th2 cells by producing IL-4 and IL-13, thereby mediating the subsequent adaptive immune response to severe infections (Gordon, 2007; Murray and Wynn, 2011; Wynn et al., 2013).

### Roles of macrophages in fracture healing

In recent years, the role of inflammatory cells in wound healing has gradually attracted great interest in related research. Macrophages play a critical and dynamic role in the process of bone regeneration and new bone formation. Bone fractures result in the disruption of local vascularity, as well as tissue integrity, inducing hematoma formation, followed by acute inflammation and the recruitment of a series of inflammatory cells. It is at this stage that monocytes and macrophages are recruited to the bone tissue (Loi et al., 2016a). In addition to these recruited macrophages, “osteal macrophages” represent another population of resident macrophages in bone tissue, which were first characterized in 2008 by Chang et al. (2008). Moreover, macrophages can differentiate into osteoclasts, which play a role in regulating the balance of the bone microenvironment by removing excess matrix (Yao et al., 2021). During the second osteogenic phase, bone resident macrophages provide important pro-anabolic support to osteoblasts and induce matrix mineralization to maintain bone homeostasis and repair. Moreover, some cytokines secreted by macrophages (e.g., IL-1β and oncostatin M) also have significant effects in terms of inducing osteoblast differentiation and bone mineralization (Lange et al., 2010; Guihard et al., 2015). TNF-α released from macrophages can increase the chemotactic ability of osteoblasts (Sun et al., 2018). Alexander et al. demonstrated for the first time that osteomacs were an integral cellular component of osteal tissues during bone healing. They indicated that osteomacs were critical participants in intramembranous ossification during bone repair (Alexander et al., 2011). Macrophages have also been increasingly recognized to play a central role in osteogenesis during the last stage of bone remodeling (Marsell and Einhorn, 2011). BMP-2 released by macrophages plays an important role in osteogenesis through Wnt and Wnt/LRP5 signaling cascades (Rawadi et al., 2003; Jamalpoor et al., 2018). Exosomes derived from M2 macrophages can promote bone formation, decrease adipogenesis, and increase miR-690 levels in bone marrow-derived mesenchymal stem cells (BMSCs), which could upregulate osteogenic differentiation (Yu et al., 2016; Li Z. et al., 2021). Dynamically controlling the regulation of macrophage phenotypes may become a new therapeutic strategy to promote fracture healing. For bone fracture healing, M1 macrophages play an important proinflammatory role in the early stages of injury, helping to resist invasion by bacteria, viruses and other pathogens, promoting antigen presentation, activating immune responses and initiating tissue regeneration; however, prolonged proinflammatory responses may delay healing (Loi et al., 2016a). M2 macrophages have been increasingly recognized as positive regulators of bone formation during fracture healing (Xiong et al., 2020); they secrete growth factors (such as platelet-derived growth factor (PDGF) and VEGF) and enzymes to promote angiogenesis and fracture healing (Niu et al., 2021). Schlundt et al. showed that increasing the proportion of M2 macrophages through IL-4 and IL-13 can significantly enhance bone regeneration (Schlundt et al., 2018). M1 macrophages preferentially infiltrate the fracture site in the acute phase, whereas M2 macrophages appear in the woven bone formation phase (Schlundt et al., 2018). It has been shown that proinflammatory factors (e.g., TNF-α, IL-6, and IFN-γ) produced by M1 macrophages inhibit osteoblast differentiation, thereby hindering collagen production by osteoblasts, which is necessary for mineralization (Abdelmagid et al., 2015). On the other hand, M2 macrophages inhibit osteoclast formation through sustained expression of TGF-β and IL-10 (Abdelmagid et al., 2015). Thus, the conversion of proinflammatory M1 macrophages into anti-inflammatory M2 macrophages is a key factor in bone healing. Several studies have shown that M1 and M2 macrophages affect the osteogenic differentiation of MSCs in different ways during fracture healing and that M1 macrophage levels are increased in early and mid-stage osteogenesis but do not enhance matrix mineralization (Omar et al., 2011; Gong et al., 2016; Zhang et al., 2017). In contrast, it has been shown that macrophage polarization to the M2 phenotype can induce preosteoblast differentiation and increase bone mineralization (Gong et al., 2016; Zhang et al., 2017), which could provide a new therapeutic target for preventing tissue damage caused by chronic inflammation (Lawrence and Natoli, 2011).

Regarding fractures, macrophages act as apex regulatory cells, with specific subpopulations sensing instructions from multiple inputs throughout the healing time frame and regulating the participation of multiple cells through these instructions. MSCs are derived from human bone marrow and are involved in intramembranous ossification, which contributes to the primary process of fracture repair. MSCs can migrate to the site of injury and then differentiate into osteoblasts, which subsequently secrete ECM proteins, including type I collagen, proteoglycans, and gamma-carboxy proteins that promote mineralization and fibrocartilage formation by increasing the polarization of M2 macrophages through BMSC-derived exosomes (BMSC-Exos) (Loi et al., 2016a; Shi et al., 2020). Autologous and allogeneic MSCs have been shown to significantly suppress the production of the inflammatory cytokine TNF-α, a process that seems to be mediated by PGE2 (Maggini et al., 2010; Ylöstalo et al., 2012; Asami et al., 2013). Bone is a highly dynamic organ whose structural integrity is maintained through precise remodeling, including osteoclasts, osteoblasts and osteocytes. Osteoclast differentiation is modulated by both macrophage colony-stimulating factor (M-CSF) and receptor activator of nuclear factor-kappa B (RANK) ligand (RANKL). Macrophages can regulate RANKL-induced osteoclastogenesis. When exposed to RANKL, osteoclast precursor cells fuse and develop the functions of active osteoclasts (Khosla, 2001). In addition, macrophages can promote or inhibit osteoclast activity by secreting TNF-α, IL-1, IL-6 and IFN-γ (Takahashi et al., 1986). IFN-γ secreted by activated T cells and macrophages has been shown to inhibit osteoclast formation by the subsequent rapid degradation of TRAF6 (Takayanagi et al., 2000). Macrophages can also indirectly promote fracture healing by secreting TNF-α, IL-1, IL-6 and CCL2 and regulating the activation of normal T cells to stimulate interstitial migration (Kon et al., 2001; Anton et al., 2012). Previous studies have shown that LPS-stimulated or IL-4-stimulated monocytes can deliver progenitor bone signals to allogeneic MSCs. Since IL-4 stimulation primarily induces Th2 responses, this study indirectly demonstrates that Th2 responses promote bone healing (Omar et al., 2011). Previous studies have shown that T cells and B cells can regulate bone resorption through osteoclasts or promote osteogenesis through osteoblasts (Croes et al., 2016; Grčević et al., 2021). During the inflammatory response phase, T cells respond to local levels of inflammatory factors to produce TNF-α (Konnecke et al., 2014). The role of TNF-α in osteogenesis is quite controversial. While several studies have suggested that TNF-α negatively regulates osteoblast differentiation (Abuna et al., 2016; Zuo et al., 2018; Constanze et al., 2020), several other studies have demonstrated quite different results. Huang et al. showed that the effect of TNF-α on osteoblast differentiation appeared to be dose dependent, with lower concentrations of TNF-α moderately enhancing the expression levels of osteogenic transcription factors and bone marker genes and higher concentrations of TNF-α exerting an inhibitory effect on osteogenic differentiation (Huang et al., 2011). Glass et al. demonstrated that TNF-α promotes fracture epithelium-mediated osteogenic differentiation of muscle-derived stromal cells (MDSCs) (Glass et al., 2011). In addition, the inhibition or stimulation of osteoblastogenesis by TNF-α seems to be associated with the differentiation lineage of the responding cells (Osta et al., 2014). T cells may also induce B cells to secrete TNF-α, which influences fracture healing (Konnecke et al., 2014). Furthermore, B cells are the main source of osteoprotegerin (OPG), which acts synergistically with T cells in the resting state to promote OPG production and protect bone (Weitzmann, 2017). Neutrophils are phagocytic cells of the myeloid lineage that are recruited by IL-1 and TNF-α secreted by platelets (Fuchs et al., 2016; Taraballi et al., 2016). Previous studies have shown that the phagocytosis of apoptotic neutrophils can also modulate the macrophage phenotype from proinflammatory to anti-inflammatory, which may support the role of neutrophils in fracture healing (Ferracini et al., 2013).

### Roles of macrophages in the elderly HF population

Macrophages are involved in multiple stages of HF healing. The characteristic immune changes that occur after HF in elderly individuals may be related to immune senescence, which refers to the aging-related decrease in the capacity of the immune system. Immunosenescence affects different tissues and cells and has unpredictable effects on the entire immune system (innate and adaptive immune responses are affected by aging) (Fuentes et al., 2017), altering the ability of the organism to adequately respond to pathogens and age-related changes in the immune response. In addition, HF often results in deep activation of the immune system. The balance of the patient’s immune system is disturbed by a significant increase in susceptibility to infection and sepsis and possibly even the development of systemic inflammatory response syndrome (SIRS), which leads to “second strike” events such as shock, infection, sepsis, and multiorgan dysfunction (Angele and Faist, 2002; Lenz et al., 2007). This inflammatory response is usually mediated by a large influx of immune cells at the cellular level at the site of injury rather than a single infectious trigger (Lord et al., 2014). As previously mentioned, macrophages exhibit great plasticity depending on the microenvironment and the different stimuli they receive and play a key role in maintaining tissue homeostasis. However, multiple functions of macrophages (including chemokine secretion and phagocytosis) are negatively altered with aging, which may cause wound infection and inhibit wound healing. We speculate that dysregulation and secondary infections due to various complications resulting from macrophage senescence may be the main cause of high mortality among elderly HF patients. Therefore, reducing mortality due to HF in elderly individuals has aroused great interest in recent years (de Miguel Artal et al., 2018; Fujita et al., 2022).

Loi et al. found that the greater alkaline phosphatase (ALP) activity and matrix mineralization of preosteoblastic cells could be significantly enhanced by coculture with macrophages modulated to the M2 phenotype in the presence of IL-4 (Loi et al., 2016b), indicating the influences of the different macrophage phenotypes under conditions of biologically compromised healing, including age. However, disruption of the M1/M2 phenotype balance often occurs in aged individuals. Gibon et al. showed that macrophages isolated from aged mice were quite different from those isolated from young animals, with a higher proportion of cells in a preactivated resting state and increased expression of the proinflammatory cytokine TNF-α (Gibon et al., 2016a). In addition, old macrophages produce fewer factors that promote the osteoblast differentiation of young bone marrow stromal cells, thus slowing bone healing in elderly mice (Vi et al., 2018). M2 macrophages are highly proangiogenic and secrete various growth factors, such as TGF-β, PDGF, and VEGF (Schlundt et al., 2018). The compromise of M2 macrophage functions by biological aging conditions could significantly influence the revascularization and matrix maturation of callus tissue during bone healing. As aging continues, circulating proinflammatory cytokines are upregulated, and this process can be described as “inflammaging.” Thus, dysregulated chemokine and cytokine expression can complicate the status of inflammaging and stress-induced immune imbalance in elderly HF patients (Franceschi et al., 2000).

**FIGURE 1:**
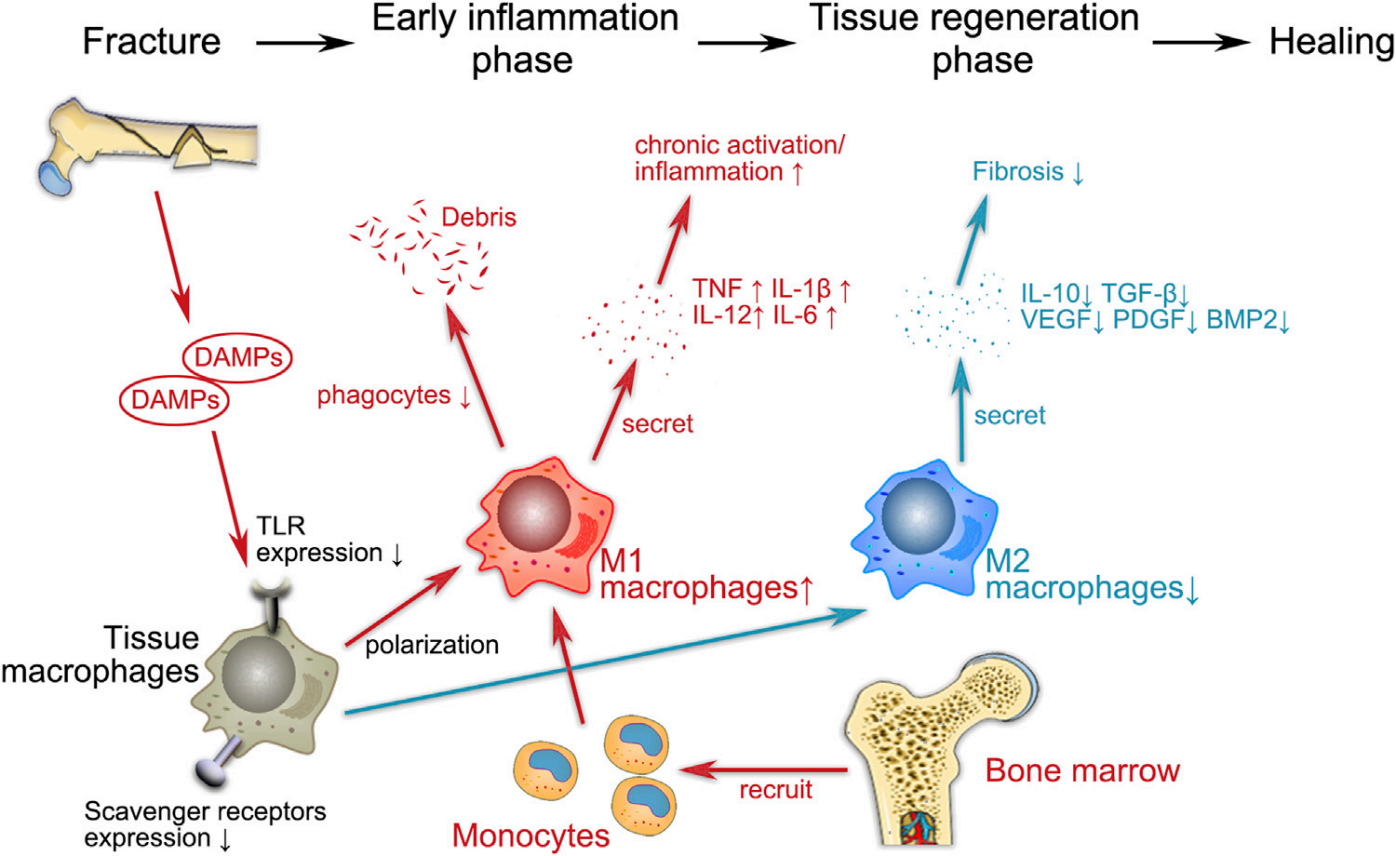
Schematic representation of the possible regulatory functions of macrophages in geriatric HF. The entire fracture healing process can be broadly divided into two phases, i.e., the early inflammatory phase and the tissue regeneration phase, during which macrophages both play a proinflammatory role and regulate subsequent fracture repair. Fractures lead to the release of endogenous cellular DAMPs, which activate macrophages via TLR signaling. During the inflammatory phase, M1 (proinflammatory) macrophages recruit monocytes from the bone marrow to the site of injury and participate in the immune response by phagocytosing cellular debris and secreting proinflammatory cytokines (e.g., TNF, IL-1β, IL-6 and IL-12). M1 macrophages promote osteogenesis in the early and middle stages without enhancing matrix mineralization. In the late repair phase, M2 (anti-inflammatory) macrophages release regenerative cytokines such as IL-10, TGF-β, BMP-2 and VEGF to establish an anti-inflammatory environment that promotes osteochondral differentiation and angiogenesis. The disruption of the immune system associated with aging inevitably has an impact on various aspects of macrophage activity (e.g., cytokine secretion, polarization, phagocytosis), which indirectly affects the entire process of fracture healing.

As the crucial role of macrophages in bone repair has become more apparent, relevant therapeutic options to promote fracture healing have emerged. Targeting the macrophage-MSC pathway to enhance bone formation now appears to be a mainstream therapeutic strategy. Local injection of M-CSF, which is a major macrophage growth factor, at the time of injury significantly increases the number of macrophages at the fracture site and subsequently improves intramembranous and intrachondral bone formation (Alexander et al., 2011; Raggatt et al., 2014). In addition, regulation of local macrophage polarization may also help bone formation. Macrophage polarization can be modulated toward an anti-inflammatory and regenerative M2 phenotype either pharmacologically or by utilizing bioactive materials (Pajarinen et al., 2019). Recently, Mahon et al. demonstrated that nanoparticle-mediated M2 macrophage polarization enhanced bone formation and MSC osteogenesis in an IL-10-dependent manner (Mahon et al., 2020). Moreover, it has been shown that local injection of the major macrophage growth factor M-CSF can significantly increase the number of macrophages at the fracture site, thereby improving intramembranous and intrachondral bone formation (Alexander et al., 2011; Raggatt et al., 2014). Therefore, it may be possible to find ways to improve declining immune malfunction in elderly individuals and thus reduce mortality due to HF in elderly individuals. A schematic representation of the possible roles and functions of macrophages is shown in Figure 1 and table1, respectively.

**TABLE 1:** Role of monocyte/macrophage in geriatric hip fracture.

Key events during fracture healing	Role of macrophage	Macrophages in geriatric hip fractures
Acute inflammation	Monocytes/macrophages are activated by inflammatory factors (e.g.IL-6, CCL-2)	Aged macrophages are less responsive to GMCSF, leading to reduced proliferation
		Monocytes/macrophages are recruited by inflammatory factors
	Clearance of necrotic tissue and provisional matrix	Phagocytosis of necrotic cells and stroma, but phagocytosis of apoptotic cells by macrophages decreases with aging
		Macrophages in the elderly are biased towards a more pro-inflammatory phenotype (M1), which is detrimental to fracture healing
	M1 macrophages secrete proinflammatory cytokines (TNF-α, IL-1β, etc.) and chemokines (CCL2, MIP-1α, etc.), which results in tissue damage with additional leukocyte infiltration.	M1 macrophage increase leads to dysregulation of inflammatory factor secretion.
		Aging leads to a decrease in the expression of anti-inflammatory cytokines, such as IL-10, by M2 macrophages.
	M2 macrophages are capable of modulating and terminating the inflammatory response	
	Activated macrophages secrete chemokines CCL2, CXCL8, and SDF-1 to recruit and activate MSCs, osteoprogenitors, and fibroblasts	Elevated systemic levels of TNFα negatively affect angiogenesis during fracture healing
		Abnormally elevated serum IL-6 levels after fracture are associated with decreased lower limb function
	Secretion of VEGF and MMP by M2 macrophages is essential for angiogenesis	Reduced secretion of growth factors TGFβ, VEGF and PDGF by M2 macrophages and delayed fracture healing
Granulation Tissue and Callus formation	Macrophages secrete IL-6 to promote callus production and angiogenesis of primary cartilage at the site of injury	The overall decreased expression of monocyte/macrophage markers CD14 and CD68 was associated with reduced callus angiogenesis
	Recruited MSCs and periosteum bone progenitor cells differentiated into osteoblasts that directly arranged the braided bone	Abnormal recruitment of mesenchymal stem cells and decreased fracture healing efficiency
	TNF-α secreted by M1 macrophages plays a central role in both intramembrane and intrchondral bone formation, including mesenchymal stem cell recruitment that stimulates chondrocyte apoptosis and osteoclast recruitment	The polarization imbalance leads to sustained high levels of TNF-α, which can cause systemic damage to tissue
Remodeling	Osteoblasts, differentiated from MSCs, secrete the organic bone matrix and induce mineralization	Differentiation of MSCs into osteoblasts is affected by inflammatory senescenc
	Osteoclasts remove immature braided bone and the underlying cartilage matrix, initiating the remodeling process	Secretion of inflammatory factors by senescent macrophages leads to abnormal activation of osteoclasts and damage to osteogenesi
	OSM produced by M1 macrophages promote mineralization by MSCs and bone healing	Decreased COX-2 expression resulted in delayed bone remodeling
	Monocytes/macrophages support osteoblast differentiation and proliferation by releasing cytokines such as BMP-2, BMP-4, TGF-β1	The number of chondrocytes expressing collagen II and osteoblasts expressing osteocalcin decreased
	Macrophage polarization to M2 phenotype induce pre-osteoblast differentiation and increase bone mineralization	The ability of MSCs to undergo osteogenic differentiation decreases with agin
	M2-like macrophages stimulated by *ß* -tricalcium phosphate enhanced the mineralization of mesenchymal stem cells	Reduction of M2 type macrophages in the elderly has a dramatic effect on fracture and tissue healin

IL-6 Interleukin-6; CCL-2 C-C motif chemokine ligand 2; CXCL-8 C-X-C motif chemokine ligand-8; SDF-1 stromal-derived factor-1; OSM oncostatin M; MSCs mesenchymal stem cell; VEGF Vascular Endothelial Growth Factor; MMP matrix metalloproteinases; GMCSF granulocyte-macrophage colony-stimulating factor; BMP Bone Morphogenetic Protein; TGF transforming growth factor; PDGF platelet derived growth factor.

## Conclusions and perspectives

Macrophages play essential roles in the healing of traumatic fractures at different age stages and tissue sites. This review focused on the immune changes in elderly HF patients. Additionally, an overview of the role of macrophages in the whole process from trauma to healing in elderly individuals is provided. Due to aging, the immune system of elderly individuals undergoes significant and comprehensive changes. The adaptive macrophage system is altered in various ways with aging, leading to a significant difference in the inflammatory response, as well as the repair process, between older and younger patients after severe trauma or fracture. Importantly, the diverse spectrum of macrophages and their flexible nature need to be considered because macrophages can play both regenerative and destructive roles. The plasticity of macrophages may allow the development of interesting therapeutic approaches for HFs and various other fractures. Inflammatory cytokines (e.g., IL-10 and TGF-β) are secreted by macrophages to promote the inflammatory response after trauma. During fracture healing, MSCs and other cells work with macrophages to promote osteogenesis. Scaffolds made of various biomaterials have been applied in the surgical treatment of fractures, and studies have shown that some biomaterials can affect the polarization state of macrophages (Franz et al., 2011), representing new targets for future clinical treatments. Although the activators and downstream effectors of macrophages involved in fracture healing are well defined, the extensive spectrum and heterogeneity of macrophage subsets and their influencing factors are still poorly understood. Several new techniques (e.g., single-cell RNA-seq and cell tracking) can be used to phenotypically analyze macrophage subpopulations at the single-cell level and reveal macrophage lineage trees, offering promising prospects for the involvement of macrophages in the treatment of fractures. HFs are one of the most common fractures in elderly individuals, and these changes in the inflammatory response with aging undoubtedly increase the difficulty of treatment, as well as the financial burden on patients. Improving elderly HF patient outcomes by designing new therapies would be the ultimate goal of nearly all geriatric research, and future work in macrophage-bone biology will contribute to achieving this goal and reducing HF-related mortality.

### Limitations

The HF field is relatively limited and may not be able to provide a very comprehensive overview of other research results related to macrophages in trauma. Most of this review covers research achievements during the past 10 years, but some aspects, especially the definitions of macrophage subtypes, have changed to oppose the previous classical concepts. Therefore, some results from the latest research seem contradictory. However, we believe that recent literature is more valuable for reference; thus, readers need to understand the limitations of this review in terms of time and source.
